# Supplementary figures and images for: CHD-associated enhancers shape human cardiomyocyte lineage commitment
Source: eLife. 2023 Apr 25;12:e86206. doi: 10.7554/eLife.86206 (PMC10156167; doi:10.7554/eLife.86206)

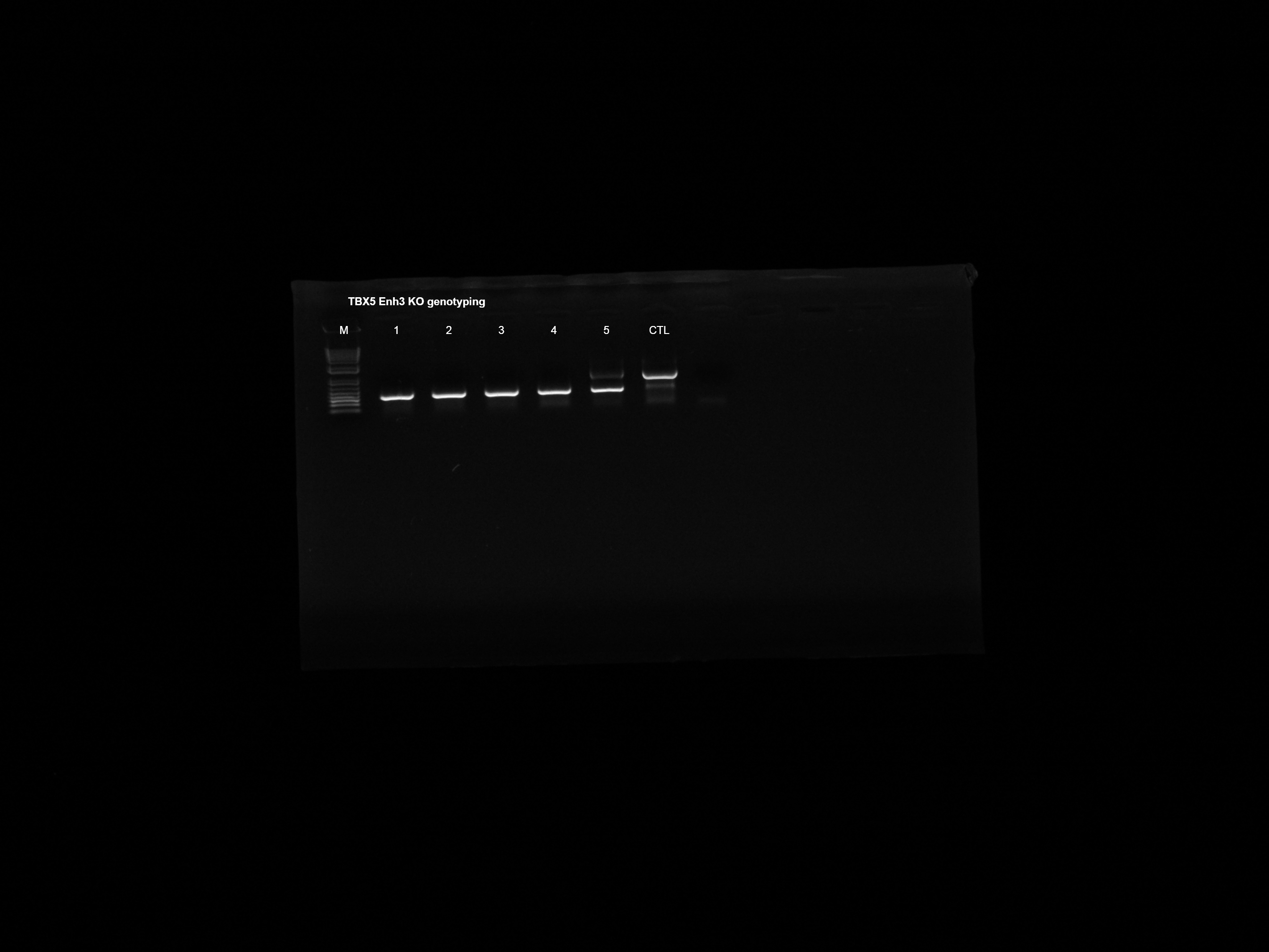

Supplement: Figure 5—source data 1. — Enhancer 3 KO genotyping gel, with labels. [file elife-86206-fig5-data1.zip › Figure 5 - source data 1.tif]

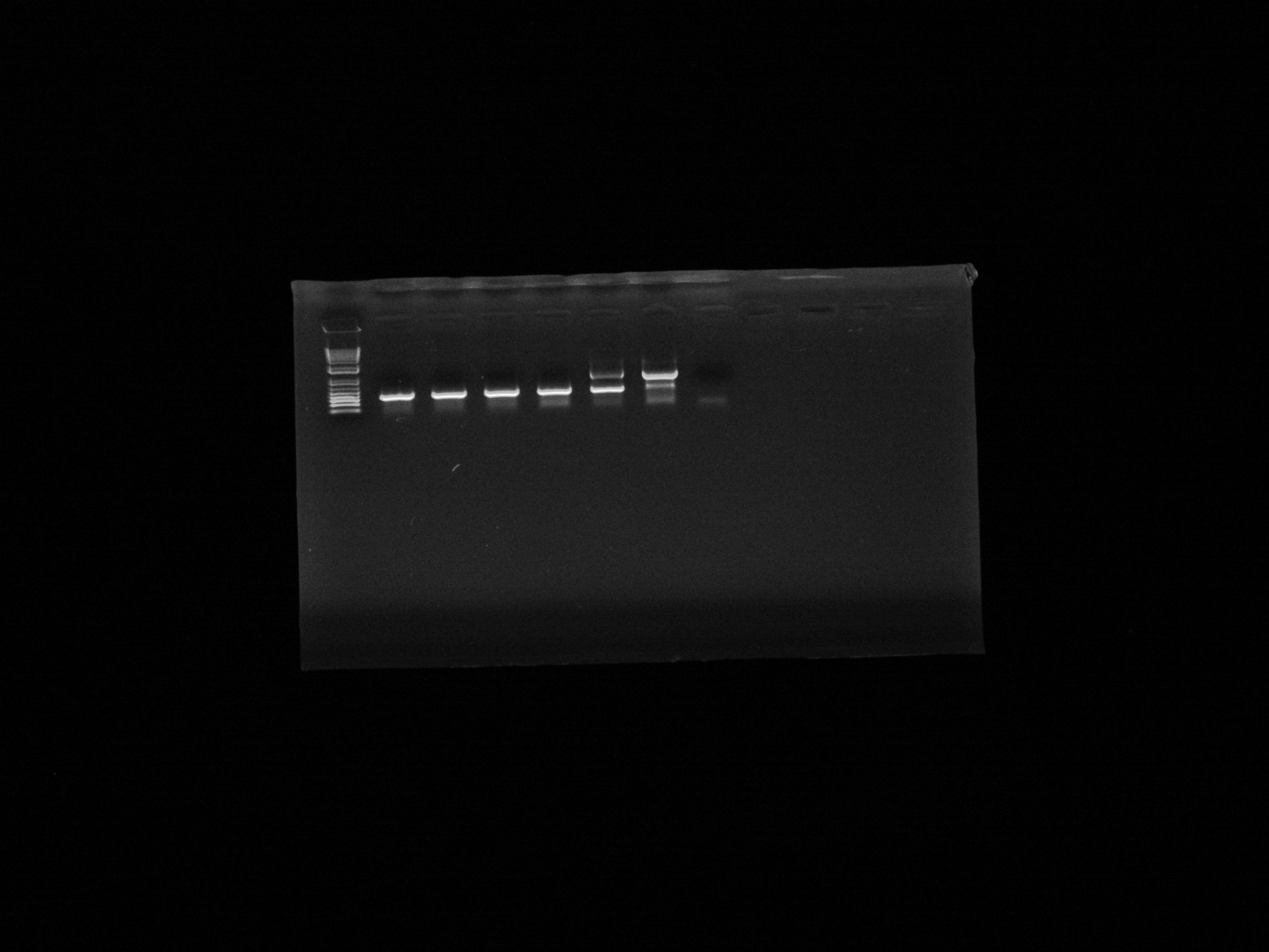

Supplement: Figure 5—source data 2. — Enhancer 3 KO genotyping gel, without labels. [file elife-86206-fig5-data2.zip › Figure 5 - source data 2.tif]

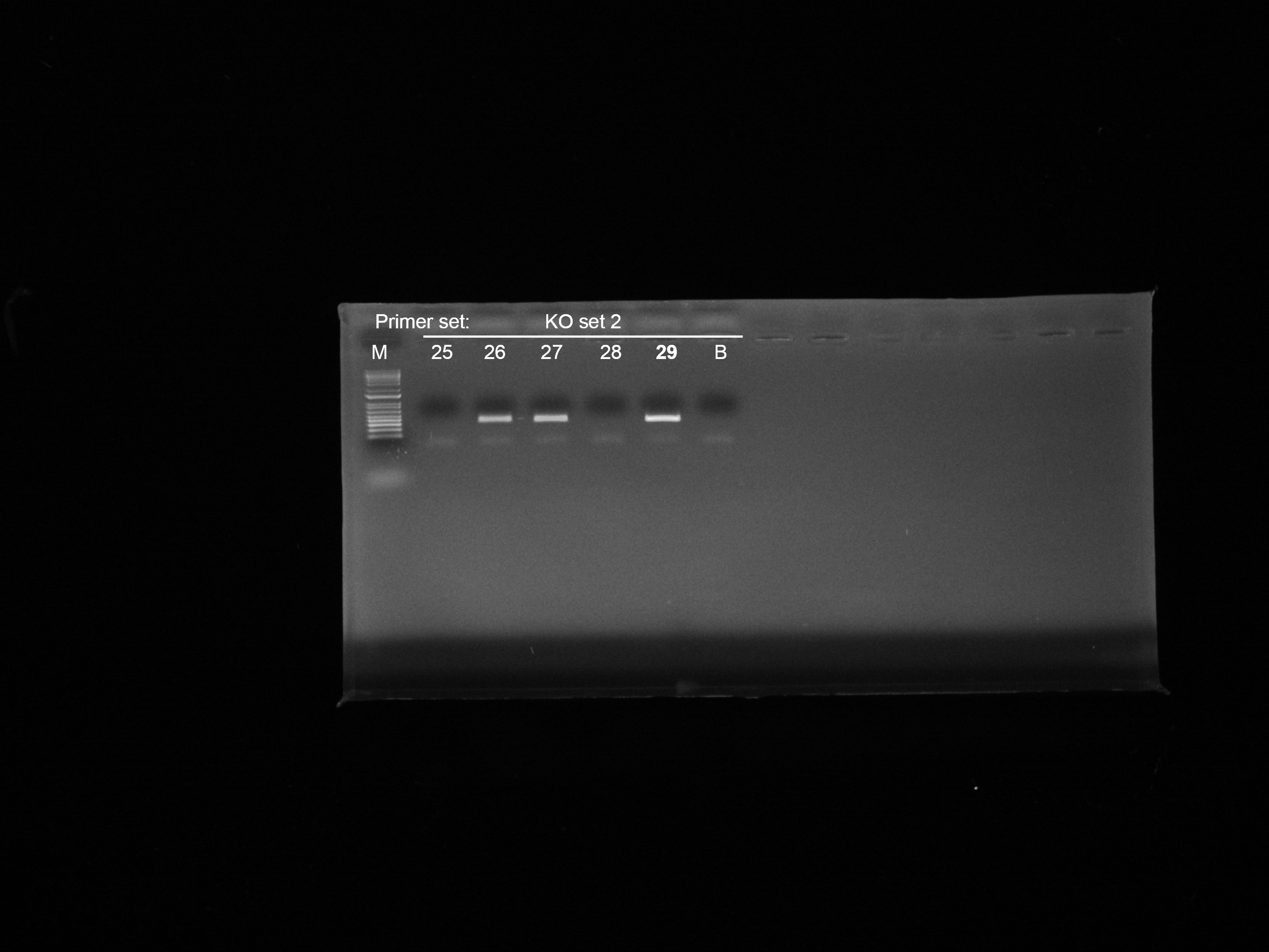

Supplement: Figure 5—source data 3. — Enhancer 5 KO genotyping gel, with labels. [file elife-86206-fig5-data3.zip › Figure 5 - source data 3.tif]

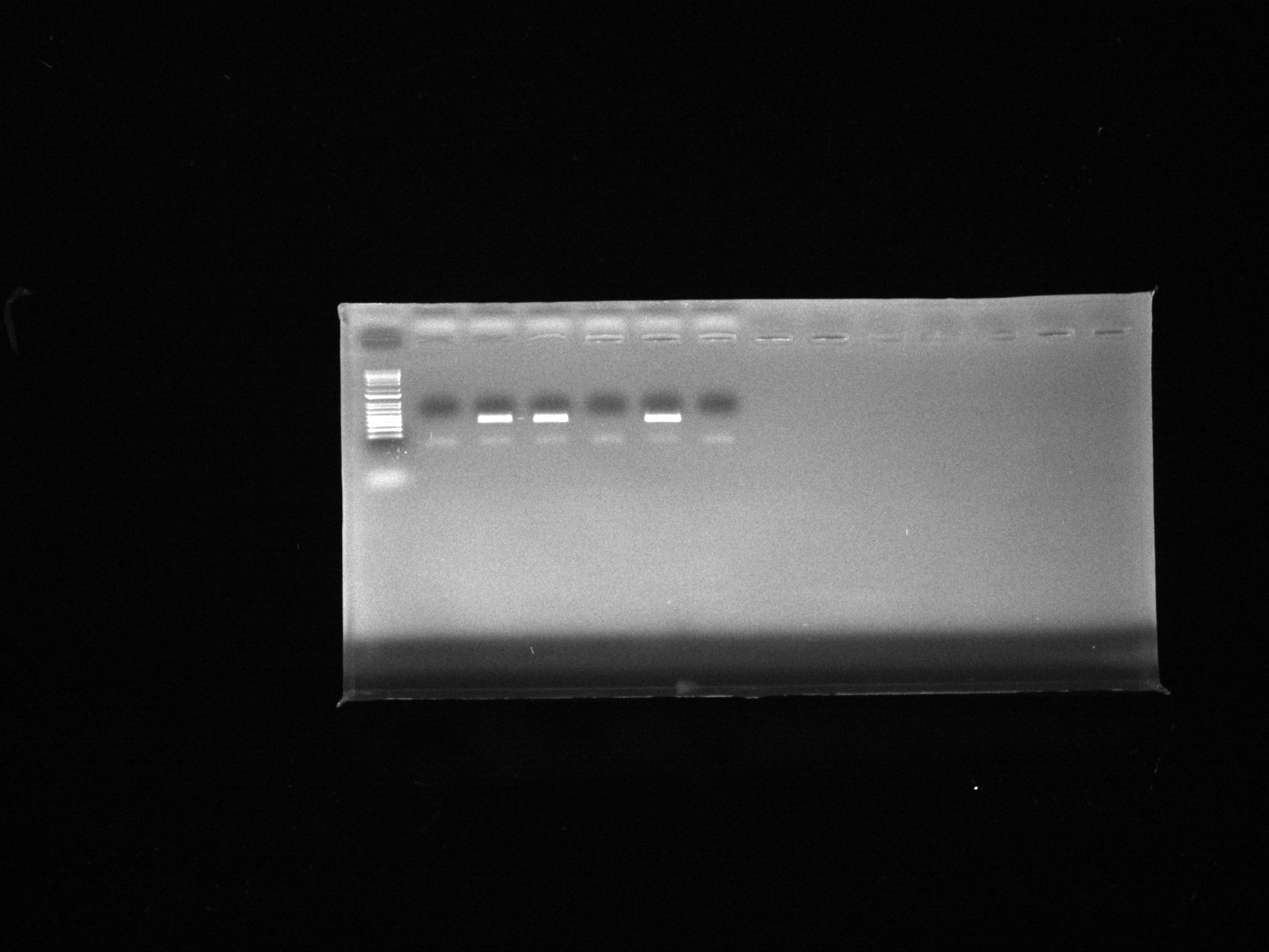

Supplement: Figure 5—source data 4. — Enhancer 5 KO genotyping gel, without labels. [file elife-86206-fig5-data4.zip › Figure 5 - source data 4.tif]

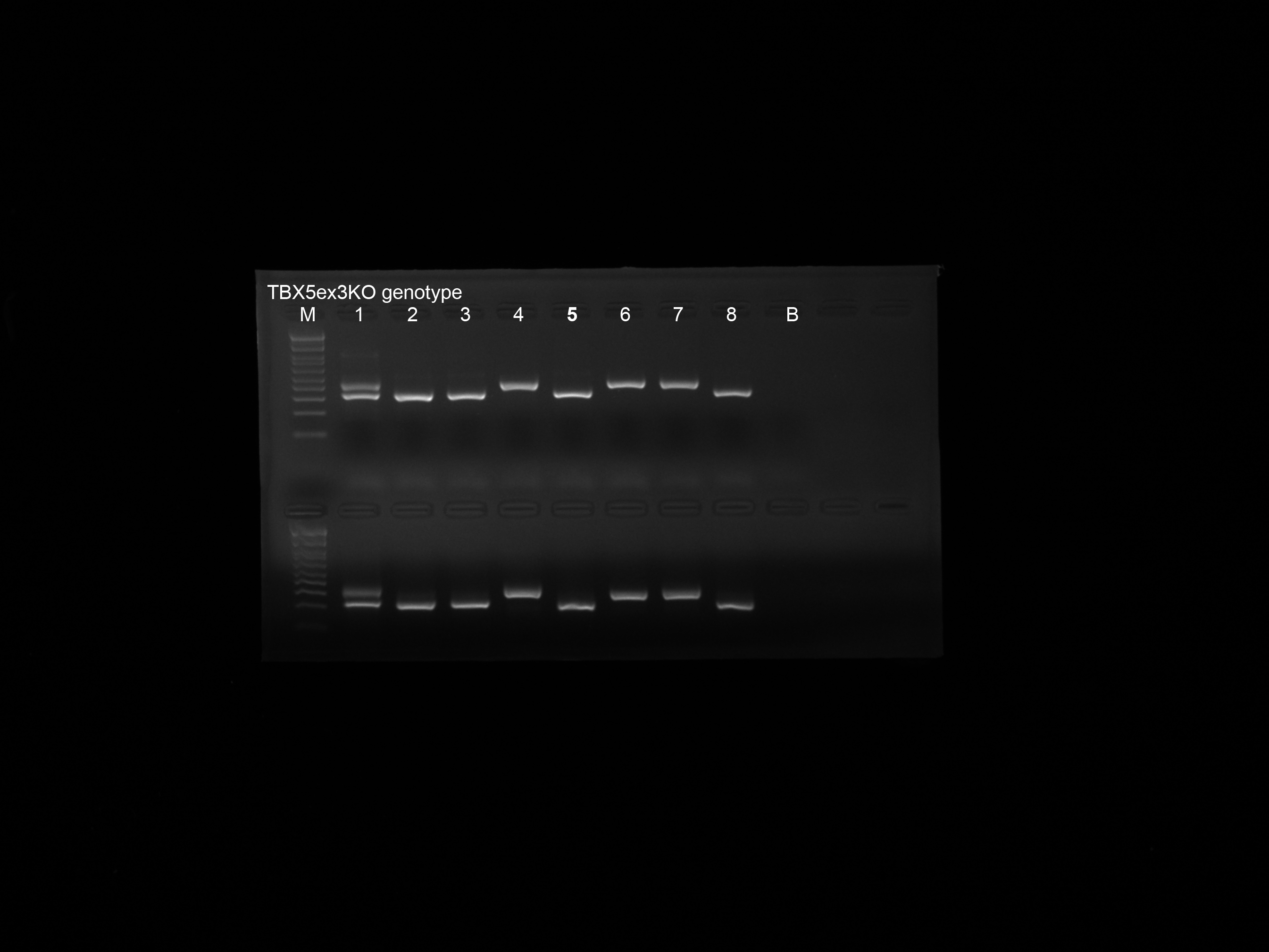

Supplement: Figure 5—source data 5. — TBX5 exon 3 KO genotyping gel, with labels. [file elife-86206-fig5-data5.zip › Figure 5 - source data 5.tif]

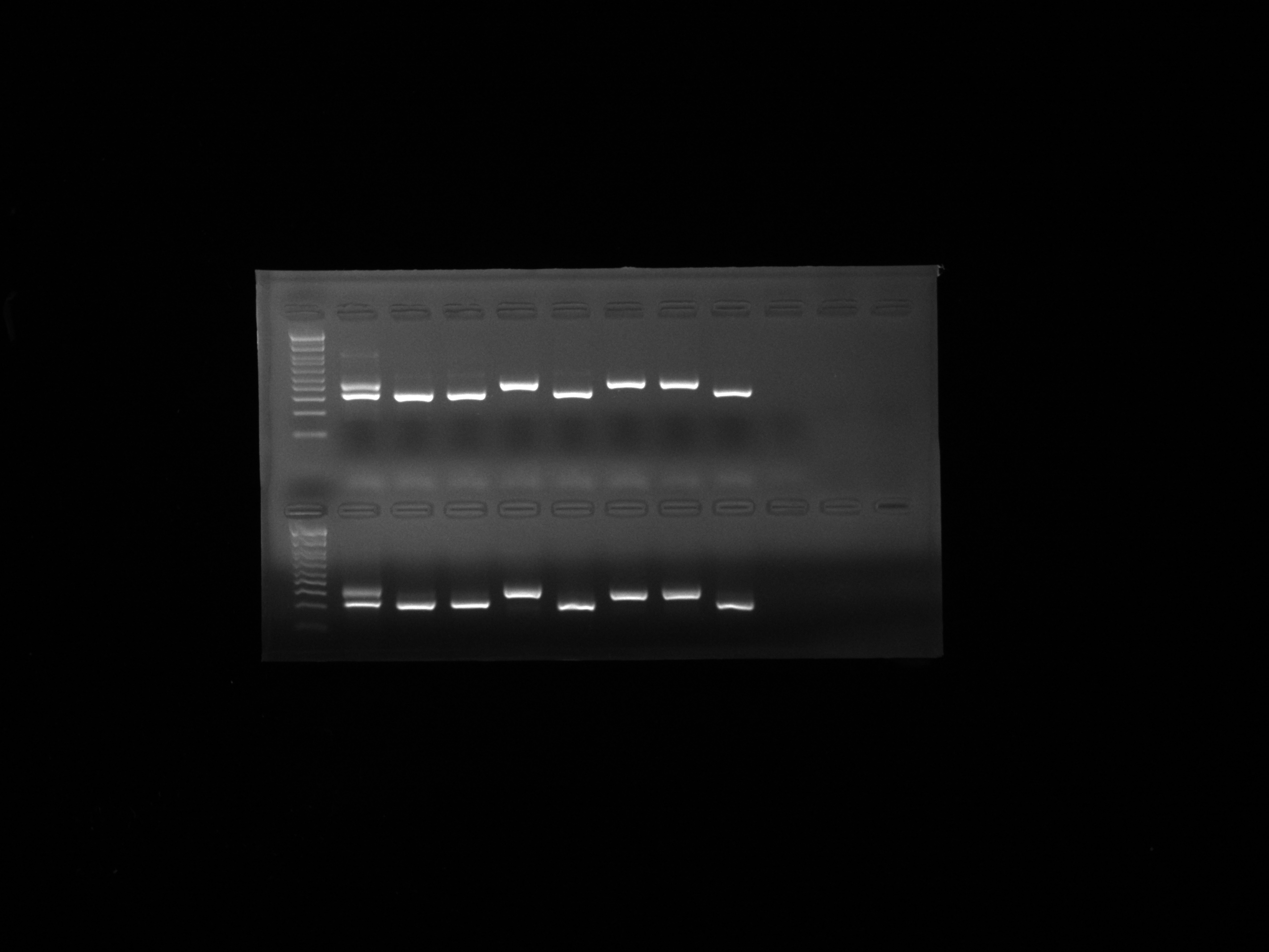

Supplement: Figure 5—source data 6. — TBX5 exon 3 KO genotyping gel, without labels. [file elife-86206-fig5-data6.zip › Figure 5 - source data 6.tif]

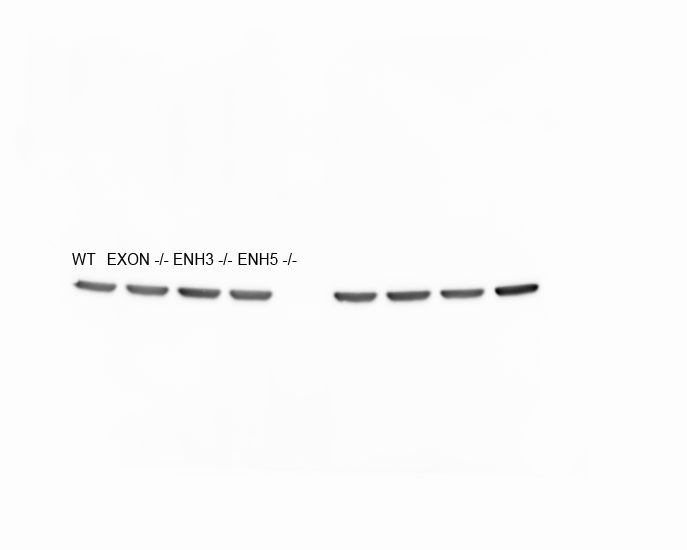

Supplement: Figure 5—source data 7. [file elife-86206-fig5-data7.zip › Figure 5 - source data 7.tif]

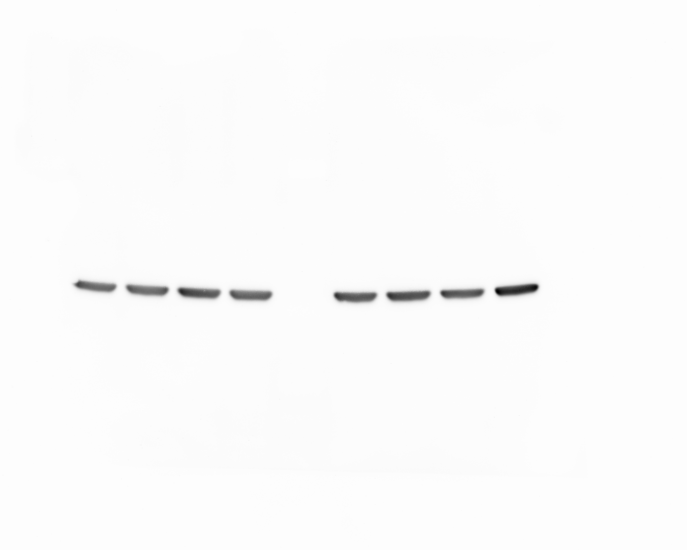

Supplement: Figure 5—source data 8. [file elife-86206-fig5-data8.zip › Figure 5 - source data 8.tif]

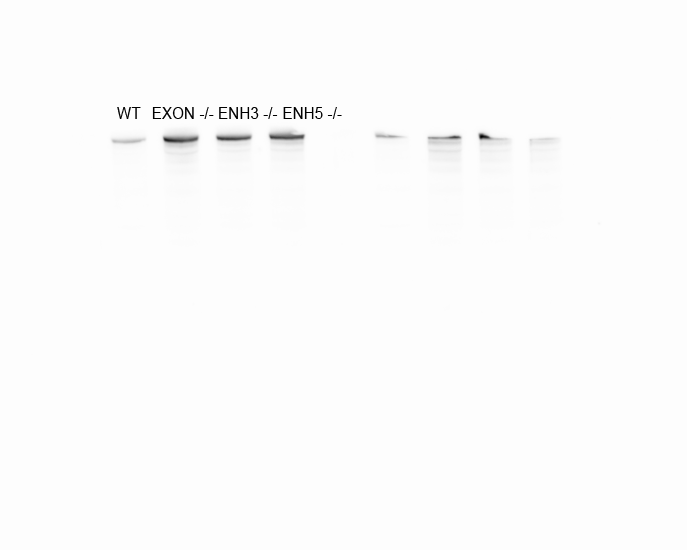

Supplement: Figure 5—source data 9. [file elife-86206-fig5-data9.zip › Figure 5 - source data 9.tif]

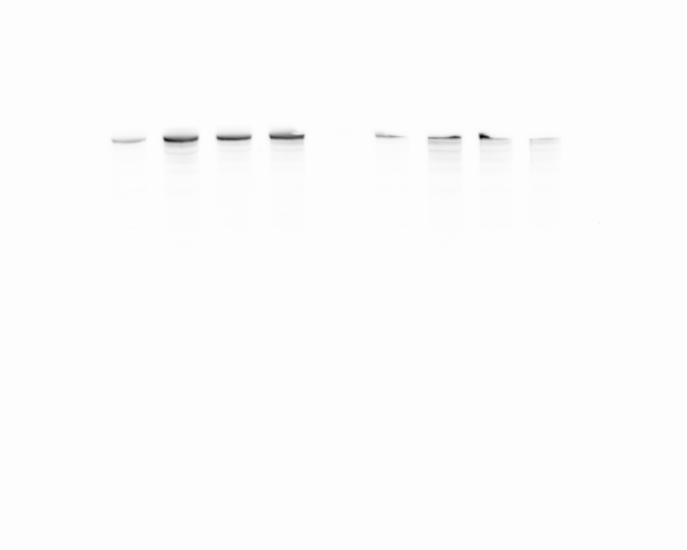

Supplement: Figure 5—source data 10. [file elife-86206-fig5-data10.zip › Figure 5 - source data 10.tif]

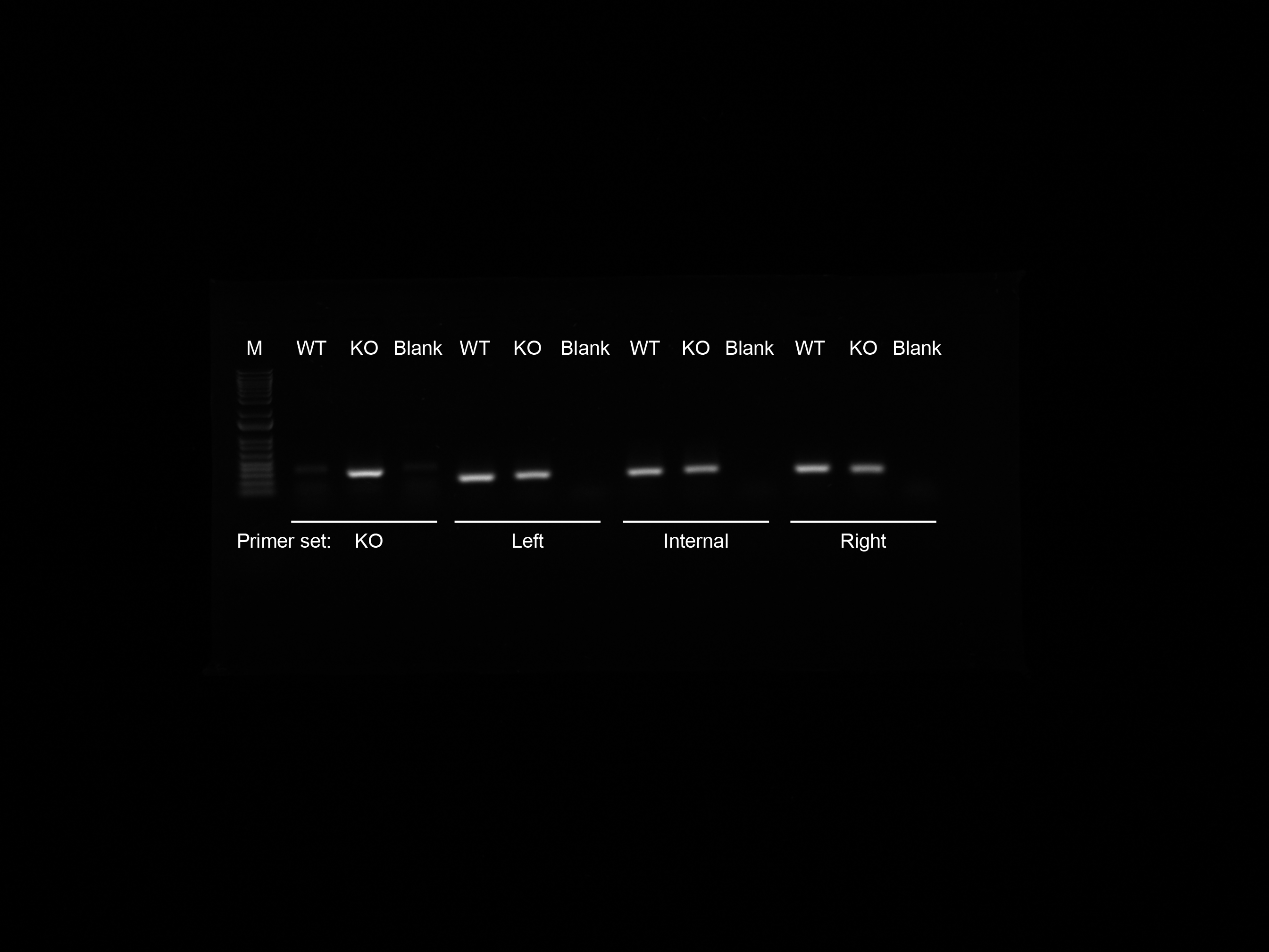

Supplement: Figure 6—source data 1. [file elife-86206-fig6-data1.zip › Figure 6 - source data 1.tif]

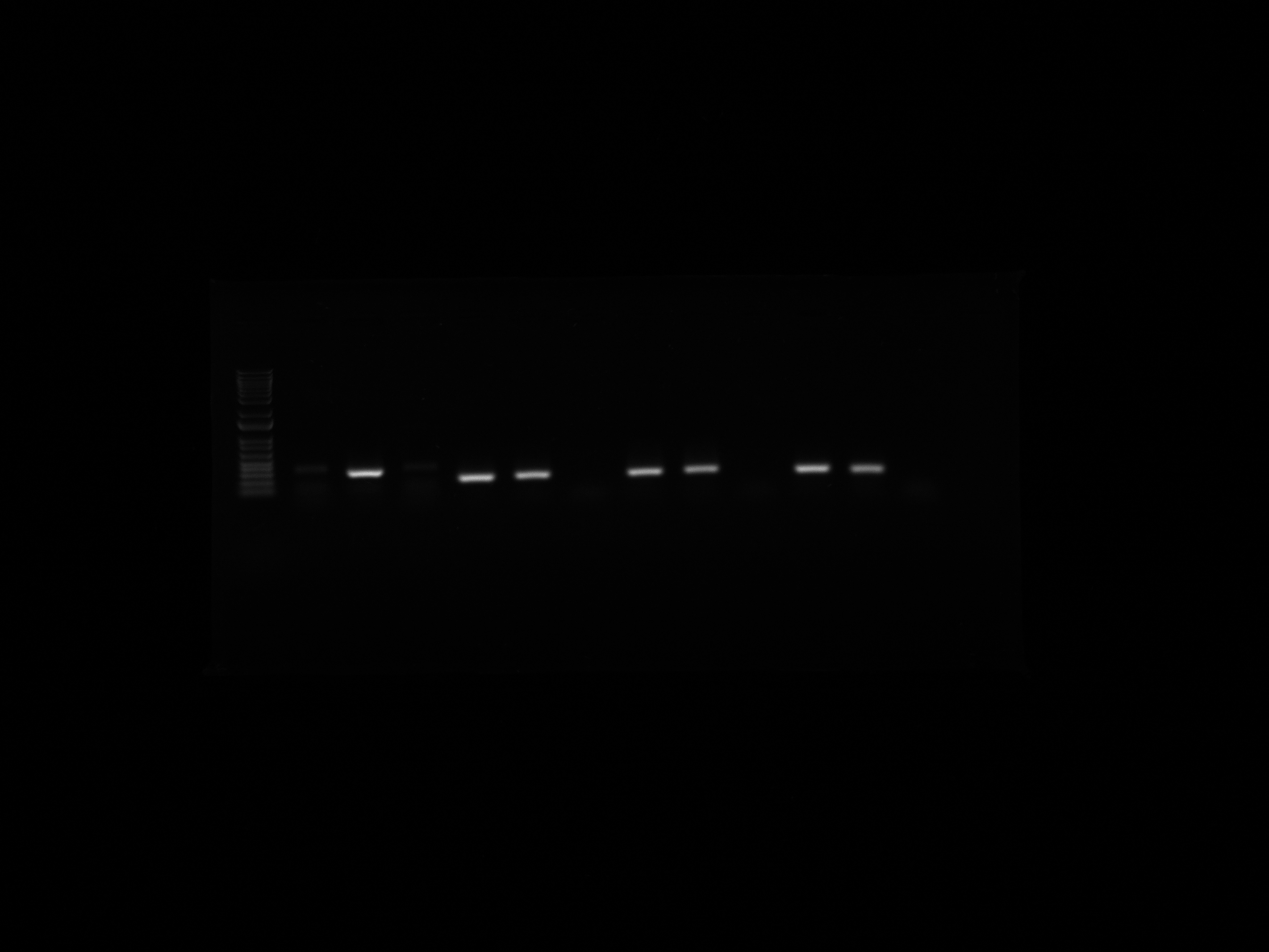

Supplement: Figure 6—source data 2. [file elife-86206-fig6-data2.zip › Figure 6 - source data 2.tif]
